# Utility of Pharmacological Agents for Diabetes Mellitus in the Prevention of Alzheimer’s Disease: Comparison of Metformin, Glucagon-Like Peptide-1 (GLP-1) Agonists, Insulin, and Sulfonylureas

**DOI:** 10.7759/cureus.87350

**Published:** 2025-07-05

**Authors:** Alexandra DiGiovanni, Andrea Shehaj, David Millar, Claire Tse, Elias Rizk

**Affiliations:** 1 Department of Neurosurgery, Penn State College of Medicine, Hershey, USA; 2 Department of Neurosurgery, University of Texas at Austin, Austin, USA

**Keywords:** alzheimer's disease, diabetes mellitus, glp-1 receptor agonist, insulin, metformin, pharmacology, sulfonylurea

## Abstract

Background: Metformin is a drug primarily used for the treatment of diabetes mellitus (DM), but it also offers clinical benefits that extend beyond glycemic control. Existing literature provides an unclear conclusion as to whether metformin's benefits extend to preventing neurodegeneration, such as in Alzheimer's disease (AD).

Methods: A retrospective database study was conducted to evaluate the likelihood of developing AD in DM patients taking metformin compared to those taking glucagon-like peptide (GLP-1) analogs, sulfonylureas, and short-acting insulin variants. An analysis was also run to assess whether metformin has a protective benefit for AD and mortality when used in those with DM compared to those without DM.

Results: In analyses totaling greater than 2.5 million patients, those on metformin had lower A1C percentages and a decreased mortality risk when compared to sulfonylureas (HR = 0.519, (CI: (0.493,0.546)), and short-acting insulins (HR = 0.372, (CI: (0.364,0.380)). Metformin use for DM was associated with a statistically significant increased likelihood of AD diagnosis compared to GLP-1 use (HR = 2.228, CI: (1.036,4.794)) but an insignificant difference compared to both sulfonylureas and insulins. Those with DM were at a significantly higher risk of being diagnosed with Alzheimer's compared to those without DM (HR = 1.826, (CI: 1.579, 2.111)).

Conclusions: Metformin, previously thought to have significant benefits in preventing neurodegeneration, may not be the optimal pharmacologic agent of choice, particularly in patients with DM, if neurodegeneration is a primary concern in treatment decision-making based on other risk factors.

## Introduction

Alzheimer's disease (AD) is a neurodegenerative condition and the leading cause of dementia worldwide [1]. Currently, AD does not have a curative therapy; this lack of disease-modifying pharmaceuticals has stimulated interest in repurposing existing and approved compounds with the potential to manage AD or delay its onset [2-4]. Metabolic disruption, such as insulin resistance, has become more prominent as a causal factor in AD development, also referred to as "type 3 diabetes" [5-7]. Given its known role in modulating cellular metabolism, metformin, a commonly administered treatment for type 2 diabetes mellitus (T2DM), has emerged as a potential pharmaceutical for decreasing the incidence and progression of AD [8,9].

Besides its hypoglycemic effect, metformin has also demonstrated protective properties in multiple other organ systems. For example, it has been shown to lower cardiovascular risk by improving endothelial function and controlling lipid metabolism [10,11]. Its anti-inflammatory and anti-cancer activity has been shown to inhibit tumor growth in various malignancies, including breast, colorectal, and endometrial cancers [12]. Metformin has also been implicated in renal protection by inhibiting fibrosis and inflammation [13]. Metformin is increasingly being investigated for its function in aging and neurodegeneration because of its many systemic actions. Recent studies have highlighted its potential for improving cognitive function and reducing AD risk [8,9,12].

Metformin primarily blocks mitochondrial complex I, a key functioning unit of the electron transport chain. Inhibition of this complex decreases adenosine triphosphate (ATP) production in cells and increases intracellular adenosine monophosphate (AMP) concentrations. This leads to the activation of adenosine monophosphate-activated protein kinase (AMPK). AMPK is an important mediator of cellular energy homeostasis, impacting mitochondrial function, oxidative stress, and autophagy [14,15]. Stimulation of AMPK in the brain has been linked to increased insulin signaling, which can oppose the neurotoxicity of insulin resistance and glucose dysregulation in AD [16]. Metformin's ability to reduce oxidative stress and inflammation also emphasizes its potential neuroprotective actions [17,18]. Nevertheless, the precise mechanisms by which metformin opposes neurodegeneration remain largely unclear in the literature.

We set out to examine the relationship between metformin and the proposed neuroprotective effects by comparing its impact on AD incidence and death with that of other commonly prescribed diabetic medications, including sulfonylureas, glucagon-like peptide-1 (GLP-1) receptor agonists, and short-acting insulin. In addition to evaluating the potential cognitive benefits of metformin, we aimed to determine whether these other diabetes medications offer similar effects.

Further, we sought to assess whether metformin has an advantage in the incidence of mortality and Alzheimer's development when used in those with a DM diagnosis compared to use in those without DM. To do this, we used a large international database called TriNetX Research Network (TriNetX, LLC, MA, USA) and examined electronic medical records to assess the effects of these therapies on AD risk and survival. We hope our results can provide insight into the impact these diabetic medications have on AD development and assist in developing future treatment protocols for these patients.

Objectives

Compare the protective value of metformin against GLP-1s, insulin, and sulfonylureas for glycated hemoglobin (HbA1c) levels, AD development, and mortality; and compare the protective utility of metformin when used in those with DM compared to when used in those without DM for HbA1c levels, AD development, and mortality. 

## Materials and methods

Data collection

The study was conducted at the Penn State College of Medicine Department of Neurosurgery in Hershey, PA. We used TriNetX, a global federated health research network that provides access to electronic medical records (diagnoses, procedures, medications, laboratory values, and genomic information) from large healthcare organizations. The TriNetX platform only uses aggregated counts and statistical summaries of de-identified information. No protected health information (PHI) or personal data is made available to the users of the platform. The data used in this study were collected on June 1st and 2nd, 2025, from the TriNetX Research Network, which provided access to de-identified information from over three million patients spanning across 100 healthcare organizations. The platform is compliant with the Health Insurance Portability and Accountability Act (HIPAA) and the General Data Protection Regulation (GDPR) regulations, and a formal consent process was not dictated for this study. TriNetX uses query cohorts that rely neither on direct nor indirect patient identifiers, and the cohorts are aggregated to further ensure privacy. The analyses were performed within the TriNetX browser-based analytics environment over the last 20 years. This study was deemed exempt from institutional review board (IRB) oversight, as the data are de-identified according to §164.514(a) of the HIPAA Privacy Rule.

Study population

Data was collected from the TriNetX information network to explore the incidence of AD and mortality in patients who were documented to have taken metformin compared to sulfonylureas, short-acting insulin, and GLP-1 analogs in individual queries. Each query in the TriNetX database was generated by including a metformin cohort of patients older than 50 years old and excluding other DM medications for each conducted analysis. A comparison between those being treated with metformin who have a diagnosis of DM, compared to those being treated with metformin without a diagnosis of DM, was conducted as well. 

Outcome variables

The data gathered was utilized in a comparative outcomes analysis generated through the TriNetX platform for AD and patient mortality outcomes (Appendix 1). The outcomes of the two cohorts were analyzed against each other in four sets. Cohort A1, metformin, as compared to Cohort A2, named insulin. Cohort B1, named metformin, was compared to Cohort B2, named sulfonylureas. Cohort C1, named metformin, was compared to Cohort C2, named GLP-1 analogs. Cohort D1, named metformin with DM, was compared to Cohort D2, named metformin without DM (DM patients excluded). For each cohort, only the DM pharmacological agent being assessed was included, and any patients taking any of the others in conjunction were excluded.

The index event was defined as the earliest time point after which outcomes were analyzed. In this case, it was the occurrence of taking one of the DM pharmacological agents up to 20 years ago. Outcomes included incidences of AD and patient mortality. The valued outcomes happen in the window starting after the first occurrence of the index event. No patients in either cohort had an index event occurrence more significant than 20 years. Average HbA1c data were gathered for each patient after the five-year time window, with the reported value being the most recently acquired one. The outcome measured was the occurrence of AD and patient mortality, excluding patients with the outcome before the time window outlined above.

TriNetX reports the percentage of race, ethnicity, and gender data for patient cohorts as reported by the healthcare organizations based on self-identified information. Self-identified race, ethnicity, and gender were then balanced between the cohorts in an effort to mitigate as many confounding variables as possible (Appendix 2). Racial classifications included Alaska Native, Asian, Black, Native Hawaiian, Other, and Unknown individuals.

Data analysis 

Propensity score matching was conducted on all specified characteristics, including demographic data, to better control for confounding variables between the compared cohorts (Appendix 2). Matching was done between diagnoses of diseases of the circulatory system (ICD 100-199), disorders of lipoprotein metabolism and other lipidemias (ICD E78), and overweight, obesity, and other hyperalimentation (ICD E65-E68) [19]. Use of the most commonly prescribed statins, atorvastatin, fluvastatin, lovastatin, pitavastatin, simvastatin, and rosuvastatin, was also balanced between patient cohorts to mitigate the impact of statins, as these medications have previously been reported to be protective against AD [20]. Characteristics were evaluated using a T-test statistical analysis to determine the cohorts' differences. 

Risk ratios and hazard ratios were utilized to test for proportionality. The TriNetX platform was used for the predominant analyses: the measure of association and the HbA1c lab result distribution. The generated data sets from TriNetX were downloaded and further investigated independently. TriNetX and Microsoft Excel (Microsoft Corporation, Redmond, Washington, United States) were utilized for all study-related data analysis and data representation, and an alpha level of 0.05 was defined as the threshold for statistical significance where applicable.

## Results

Patient population demographics

**Figure 1:**
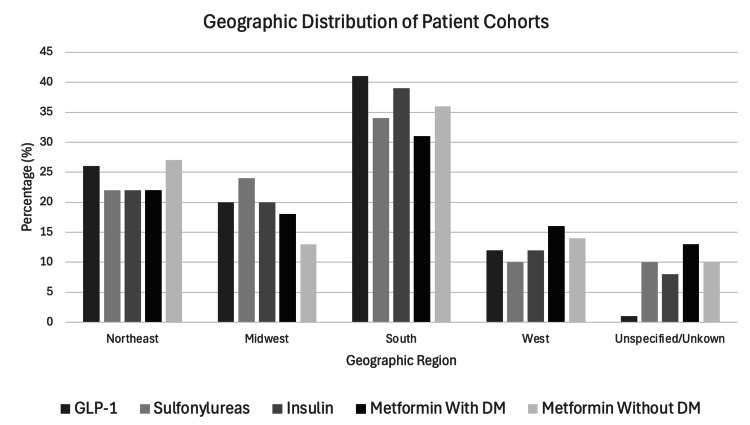
Geographic distribution (%) of each patient cohort assessed TriNetX reports geographic distribution based on the healthcare organization that reported the patient information. Northeast, Midwest, South, and West refer to regions of the United States. The unspecified and unknown category includes both those reported as Ex-US without further geographic delineation and those reported as unknown. GLP-1: glucagon-like peptide-1; DM: diabetes mellitus

Following propensity score matching analyses, A-C included two groups: the first three included patients taking metformin and excluding the other DM medication, and the second included patients taking the other DM medications but excluding metformin. Analysis D also included two groups, with the first consisting of those both with DM and on metformin and the second being those on metformin but without DM. On average, the comparison of metformin versus sulfonylureas included the oldest patients, with an average age of 69.2 years at the time of analysis. The metformin with DM versus metformin without DM analysis included the youngest patients on average, with 62.6 and 62.9 years old being the average age for those with and without DM, respectively. There was variation in sex and race across the generated cohorts as well. The metformin vs. GLP-1 analysis had the highest percentage of female patients and White patients. Baseline characteristics on mean age, sex breakdown, and race characteristics are further depicted in Table 1. TriNetX also provides geographical distributions of patients based on the location of the healthcare organization from which information was reported (Figure 1).

**Table 1:** Demographics of cohorts after propensity score matching The table includes the analysis groups compared against each other. Group A1, B1, and C1 = metformin (no glucagon-like peptide-1 (GLP-1), no sulfonylureas, and no short-acting insulins), Group A2, B2, and C2 = GLP-1, sulfonylureas, and short-acting insulins (excluding metformin). Group D1 indicates those treated with metformin for diabetes mellitus (DM), while D2 indicates those treated with metformin for a reason other than a formal DM diagnosis. The mean current age is also included as age ± SD (years). Selected race data was included; unknown race was not included in the table.

Analysis Group	Treatment Group	Mean Age ± SD (yrs)	Female	White Patients	Black Patients	Asian Patients
A: Metformin vs. GLP-1	A1: Metformin	61.8 ± 9.11	47,662 (61.9%)	50,131 (65.1%)	14,134 (18.4%)	2,348 (3.1%)
	A2: GLP-1	61.9 ± 9.02	47,560 (61.8%)	49,931 (64.9%)	14,031 (18.2%)	2,422 (3.1%)
B: Metformin vs. Sulfonylureas	B1: Metformin	69.2 ± 11.6	52,037 (47.3%)	63,073 (57.3%)	17,396 (15.8%)	5,982 (5.4%)
	B2: Sulfonylureas	69.2 ± 11.7	51,953 (47.2%)	63,067 (57.3%)	17,304 (15.7%)	5,968 (5.4%)
C: Metformin vs. Short-acting insulins	C1: Metformin	64.4 ± 10.5	368,322 (48.3%)	445,306 (58.4%)	123,969 (16.3%)	54,905 (7.2%)
	C2: Short-acting insulins	63.7 ± 11.1	367,078 (48.1%)	448,867 (58.8%)	126,531 (16.6%)	51,556 (6.8%)
D. Metformin in those with DM vs. Metformin in those without DM	D1: Metformin (+ DM)	62.6 ± 10.6	258,424 (53.9%)	276,914 (57.7%)	63,573 (13.2%)	23,065 (4.8%)
	D2: Metformin (- DM)	62.9 ± 10.8	251,520 (52.4%)	273,635 (57.0%)	64,382 (13.4%)	22,641 (4.7%)

Alzheimer's disease and mortality

There was a significantly higher risk of incidence of AD demonstrated for analysis A in those with metformin vs. GLP-1, with a hazard ratio (HR) with a 95% CI of 2.228 (1.036, 4.794). The p-value for proportionality testing was 0.53 for analysis A. There was not a statistically significant difference in risk in analysis B comparing metformin and sulfonylureas with an HR of 1.13 (0.952, 1.34) or in analysis C comparing metformin and short-acting insulin with an HR of 1.02 (0.947, 1.098).

The risk of developing AD in those with DM on metformin compared to those without DM on metformin, analysis D, was determined to be significantly higher in the former cohort, with an HR of 1.826 (1.579, 2.111), but the p-value for proportionality testing was < 0.0001 for analysis D.

Regarding mortality outcomes, analysis A indicated a statistically insignificant risk difference in mortality in those on metformin compared to GLP-1s, while analyses B and C demonstrated lower mortality risk for those on metformin compared to sulfonylureas and insulin. The hazard ratios (95% CI) of analyses A, B, and C were 1.046 (0.849, 1.289), 0.519 (0.493, 0.546), and 0.372 (0.364, 0.380), respectively. The p-values for proportionality testing were 0.12 for analysis B and < 0.0001 for analysis C. In analysis D, the HR was 0.965 (0.929, 1.003), indicating an insignificant difference in mortality risk in those on metformin with DM compared to those on metformin without DM. The patients in each cohort, patients with the outcome, hazard ratios, and their 95% confidence intervals are displayed in Table 2.

**Table 2:** Outcomes and calculated risk and hazard ratios for Alzheimer's disease and mortality The table includes the analysis groups compared against each other as it relates to the hazard ratio for various Alzheimer's disease and mortality. The treatment groups with each analysis group exclude patients who have at any time point taken the other medication (for example, in analysis A, the metformin cohort excludes patients who have taken GLP-1, and the GLP-1 group excludes patients who were recorded to have taken metformin). *Indicates a significant confidence interval. **Indicates a non-zero result of less than 10 patients that the TriNetX network automatically rounds to 10 for reporting purposes. GLP-1: glucagon-like peptide-1; DM: diabetes mellitus

Analysis Group	Outcome	Cohort	Patients in the Cohort	Patients With Outcome	Hazard Ratio	95% Confidence Interval
A: Metformin vs. GLP-1	Alzheimer's disease	Metformin	76,486	131	2.228	(1.036, 4.794)*
		GLP-1	76,720	≤ 10**		
	Mortality	Metformin	74,684	890	1.046	(0.849, 1.289)
		GLP-1	75,918	99		
B: Metformin vs. Sulfonylurea	Alzheimer's disease	Metformin	1,07,908	272	1.13	(0.952, 1.34)
		Sulfonylurea	1,07,889	257		
	Mortality	Metformin	1,03,308	2,238	0.519	(0.493, 0.546)*
		Sulfonylurea	96,748	4,509		
C: Metformin vs. Insulin	Alzheimer's disease	Metformin	7,54,398	1,522	1.02	(0.947, 1.098)
		Insulin	7,51,401	1,308		
	Mortality	Metformin	7,31,601	11,968	0.372	(0.364, 0.380)*
		Insulin	6,24,315	26,533		
D. Metformin in those with DM vs. Metformin in those without DM	Alzheimer's disease	With DM	4,75,848	692	1.826	(1.579, 2.111)*
		Without DM	4,77,733	248		
	Mortality	With DM	4,63,328	6,442	0.965	(0.929, 1.003)
		Without DM	4,64,223	4,366		

HbA1C

**Figure 2:**
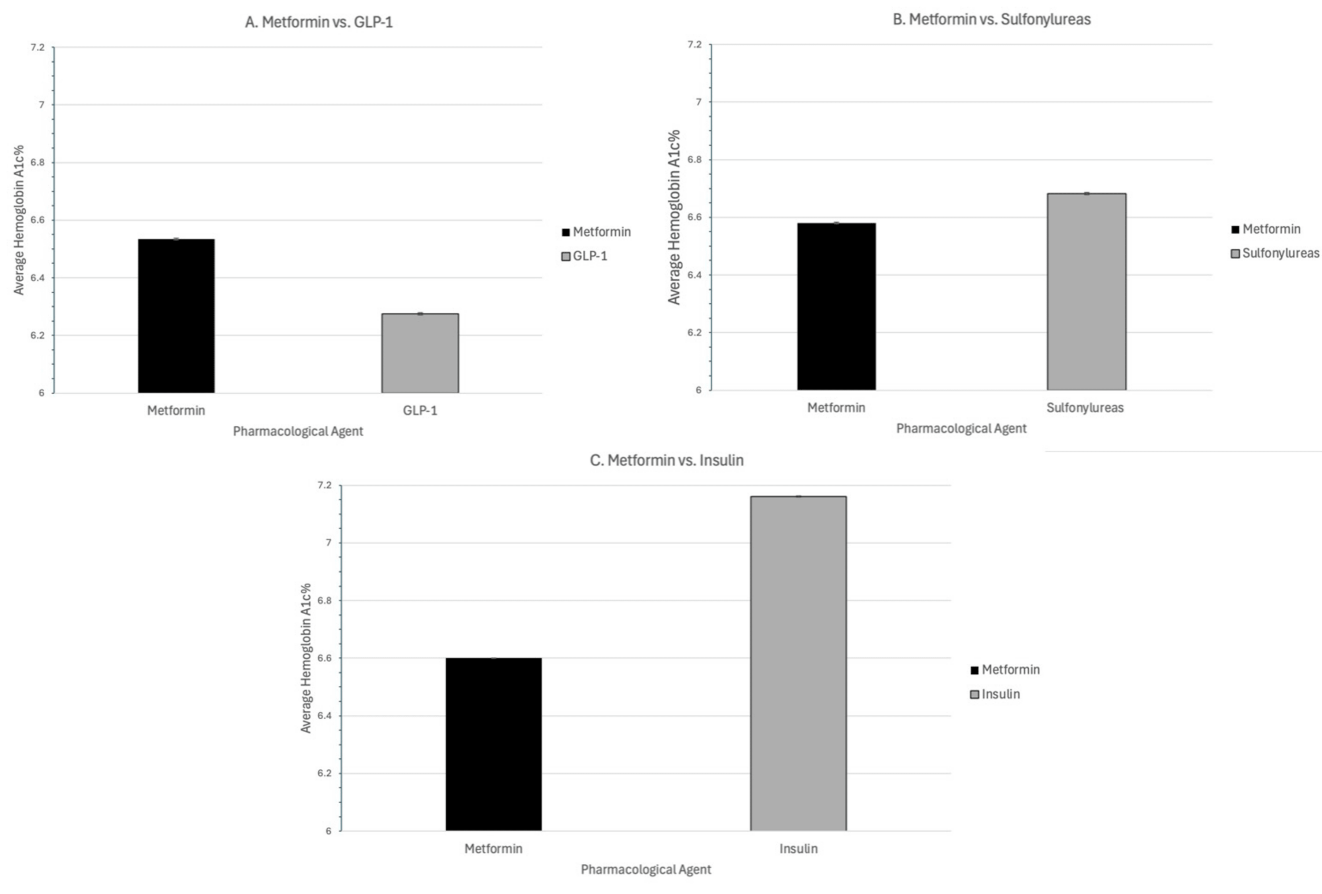
HbA1c for each cohort (A-C) A: metformin vs. GLP-1; B: metformin vs. sulfonylureas; C: metformin vs. short-acting insulin. The average HbA1c is depicted on the y-axis, and the pharmacological agent on the x-axis. HbA1c: glycated hemoglobin; GLP-1: glucagon-like peptide-1

**Figure 3:**
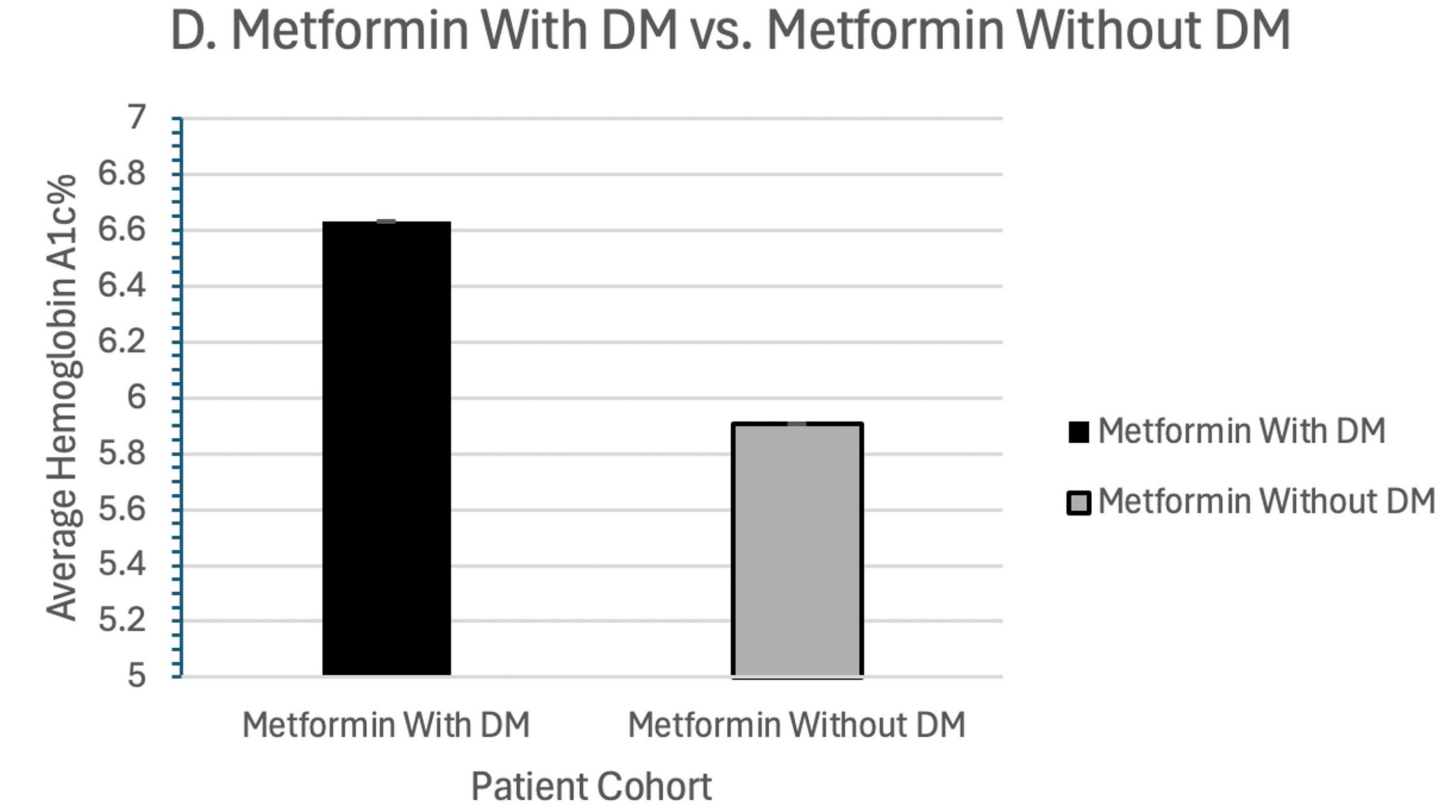
HbA1c for cohort D Metformin in patients with DM vs. metformin in patients without DM. The average HbA1c is depicted on the y-axis, and the patient cohort on the x-axis. HbA1c: glycated hemoglobin; DM: diabetes mellitus

When comparing HbA1c results in analysis A, patients only taking metformin had a mean HbA1c value of 6.53% (SD 1.05%), while patients only taking GLP-1 had a mean HbA1c value of 6.28% (SD 1.14%) (p<0.0001) (Figure 2). For analysis B, patients only taking metformin had a mean HbA1c value of 6.58% (SD 1.01%), while patients only taking sulfonylureas had a mean HbA1c value of 6.68% (SD 1.22%) (p<0.0001). In analysis C, patients only taking metformin had a mean HbA1c value of 6.60% (SD 1.05%), while patients taking only short-acting insulins had a mean HbA1c value of 7.16% (SD 1.70%) (p<0.0001). For analysis D, patients with a diagnosis of DM taking metformin had a mean HbA1c value of 6.63% (SD 1.10%), while patients taking metformin without a DM diagnosis had a mean HbA1c value of 5.91% (SD 0.80%) (p < 0.0001) (Figure 3).

## Discussion

This retrospective study of a large database investigated the effect of DM pharmacologic agents on AD incidence to assess whether metformin has a more significant protective benefit against neurodegenerative processes than GLP-1, sulfonylureas, and short-acting insulin [18,21]. Metformin's use for DM treatment is generally considered to be a first-line option due to affordability, accessibility, and effectiveness in lowering A1C, as evidenced here with patients having taken metformin having reduced values when compared to sulfonylureas and short-acting insulins with differences of 0.1% and 0.56%, respectively [22]. However, this trend did not persist when comparing it to GLP-1s, where the patient cohort had, on average, an HbA1c higher by 0.25% in those with metformin, which supports a previously conducted Danish study that found the reduction of A1c values to be greater in those using GLP-1s than in metformin [23]. 

For those taking metformin for DM compared to those taking it without a DM diagnosis but rather for another clinical indication, the HbA1C difference was 0.72%, the most marked difference compared to the other analyses, which was expected given the notably elevated levels in those with DM, coupled with the known clinical difficulty in fully mitigating the rise in HbA1C levels in those with DM. When comparing individuals with diabetes on metformin to those without diabetes, the risk of mortality was not statistically different (HR = 0.965, CI: 0.929, 1.003).

Metformin's benefit, compared to sulfonylureas and insulins, was further evidenced by the reduced mortality rates in the metformin cohort when compared to those treated with another DM pharmacologic agent. The drug is associated with improved lipoprotein levels and body weight reduction, both of which, when elevated above normal, have negative implications on rates of cardiovascular and non-cardiovascular deaths, thereby implicating metformin as a valuable protective agent for mortality benefit [24-26]. In this study, metformin had a protective benefit in mortality rate when compared to sulfonylureas (HR = 0.519, CI: 0.493, 0.546) and short-acting insulins (HR = 0.372, CI: 0.364, 0.380), and though the risk of mortality was slightly higher in those on GLP-1s than those on metformin, this difference was not statistically significant (HR = 1.046, CI: 0.849, 1.289). The observed mortality benefits further support the existing literature's claims of metformin's utility as an effective therapeutic agent for patients with DM when considering mortality outcomes. Metformin provides a mortality benefit in individuals with diabetes compared to sulfonylureas and insulin, as indicated herein with proportionality values of 0.12 and < 0.0001, respectively, suggesting that the role of time course should be further investigated for the latter relationship in particular to elucidate information comparing the risk of mortality with short- and long-term use, as this study looked at overall incidence and did not stratify cohorts based on time.

Beyond its role in glycemic control, metformin is known to exert beneficial effects across multiple organ systems by reducing the risk of various diseases. Mechanistically, it upregulates AMPK activation and therefore insulin signaling, which is hypothesized to be protective against AD. Since AD is linked to insulin resistance and glucose dysregulation, both of which contribute to neurotoxic disease progression, metformin's potential role in mitigating these effects is of significant interest [16].

In this study, however, metformin's neuroprotective effects against AD, compared to other treatments, were inconsistent. There was no comparison between analyses A-C, in which metformin demonstrated a protective effect against neurologic decline based on the incidence of an AD diagnosis. There was no statistically significant difference when compared to sulfonylureas (HR = 1.13, CI: 0.952, 1.34) or insulin (HR = 1.02, CI: 0.947, 1.098), suggesting that other factors beyond metabolic control contribute to the likelihood of AD development. Interestingly, a previous study by Che-Wuan Yu et al. provided evidence that sulfonylureas may be a suboptimal choice when compared to dipeptidyl peptidase-4 (DPP-4) inhibitors, not assessed herein, for cognitive longevity, leveraging the question as to whether expanding beyond the mainstay DM treatments alone to evaluate possible benefits of combination therapy both on metrics of diabetes control and also neurodegeneration prevention would prove useful in maximizing the utility of existing medications [21].

Despite the negligible difference in this study between AD risk with metformin compared to both sulfonylureas and insulin, patients on metformin were actually more likely to develop AD than those on GLP-1 receptor agonists (HR = 2.228, CI: 1.036, 4.794), which helps to underscore a recently published study in Nature Medicine that found GLP-1s to be protective to some degree against AD development due to several reducing impacts, including on neuroinflammation and oxidative stress levels [27]. This relationship had a proportionality value of 0.63, suggesting continuity over time.

Generally, metformin was not found to be protective against AD compared to other DM pharmacologic agents. In analysis D, comparing those on metformin with DM to those on metformin without DM, those with DM, which is known to impair glycemic control mechanisms and therefore significantly increase the risk of AD development, were at a significantly higher risk of being diagnosed with Alzheimer's (HR = 1.826, CI: 1.579, 2.111), and this relationship had a proportionality value of 0.53. This finding serves to underscore the value of continuing to explore the proper management and regulation of DM. A useful comparison to further elucidate information about metformin’s benefit would be to assess differences in Alzheimer’s and mortality risk in those on metformin but without DM, compared to those not on metformin and without DM, though the database itself did not allow for this type of cohort generation, thereby illuminating an opportunity for exploration.

As with many investigations into metformin's broader clinical applications, these findings provide both insights and avenues for further research. Continued exploration is necessary to understand the neuroprotective potential of diabetes medications fully and to optimize treatment strategies for patient outcomes. Regardless, this study expands the information base of whether these drugs and their unintended benefits can truly be mobilized to benefit patient care. Metformin, thought previously to have significant benefits in preventing neurodegeneration, may not be the pharmacologic agent of choice in all patients with DM if neurodegeneration is a primary concern in treatment decision-making based on other risk factors. This study suggests that further inquiry into alternatives, chiefly GLP-1s, may yield a meaningful pursuit as we seek to better serve patients with DM and prevent its sequelae of potential consequences, including neurodegeneration.

Limitations

The extent to which improved outcomes are a result of the ease of access and use is likely highest in metformin, thus promoting patient adherence and consistent and appropriately given treatment for the control of DM exacerbations. This is therefore a potential limitation in the utility of the results when seeking to quantify metformin's intrinsic benefit, potentially overestimating its pharmaceutical value compared to other drugs [24].

Another limitation is that TriNetX and other databases that amass large amounts of information based on diagnosis codes and outcomes have an inherent maximum threshold in research utility due to the potential for some of the assessed diagnoses or outcomes to have been recorded in a misleading or convoluted way that the database itself does not give researchers the capacity to vet. Studies that conduct similar analyses using patient-level data are needed to further confirm the findings reported here, as generalizability cannot be guaranteed, and only associations rather than causality can be determined.

Further, TriNetX cohort comparisons require inclusion criteria, so though it would have been useful to compare a cohort of patients on metformin and without DM to those not on metformin and without DM, an inclusion criterion that would have narrowed the scope would have been necessary. This can be an extension that is done using patient-level data but was not able to be effectively done herein. However, this study still provides the most recent information comparing the effect of metformin on AD incidence compared to other pharmacologic agents used in treating DM. 

## Conclusions

Metformin has been heavily investigated for its potential utility in neurodegeneration prevention because of its known protective benefits in many organ systems, besides its primarily marketed benefit of glycemic control. While metformin has long been considered to have a notable effect in terms of improving HbA1C levels and mortality outcomes, its utility in preventing AD development is limited and is not the most consistently protective compared to all its pharmacological counterparts used to treat DM. While further research on these DM pharmacologic agents is necessary to maximize their utility, this study can aid in identifying avenues for future prospective studies that aim to amass evidence that can aid in clinical decision-making regarding diabetes treatment, especially for those who are at risk of neurodegenerative processes.

## Appendices

Appendix 1 

**Table 3:** Query for cohort generation in TriNetX This table outlines the entries used in TriNetX to generate queries and subsequent cohorts. Boxes with hyphens indicate the first variable of the inclusion criteria entered and therefore do not have corresponding articles (and, or). DM: diabetes mellitus

Query Criteria for Metformin With DM				
Must have	-	Medication	NLM:RXNORM:6809	Metformin
	And	Diabetes Mellitus		
	And	Age is older than 50 years before the index event		
Cannot have	-	Medication	NLM:ATC:A10BJ	Glucagon-like peptide-1 (GLP-1) analogues
	or	Medication	NLM:HS501	Insulin
	or	Medication	NLM:ATC:A10BB	Sulfonylureas
Query Criteria for Insulin				
Must have	Any of	Medication	NLM:RXNORM:51428	Insulin aspart, human
		Medication	NLM:RXNORM:400008	Insulin glulisine, human
		Medication	NLM:RXNORM:86009	Insulin lispro
	And	Diabetes Mellitus		
	And	Age is older than 50 years before the index event		
Cannot have	-	Medication	NLM:RXNORM:6809	Metformin
	or	Medication	NLM:ATC:A10BJ	Glucagon-like peptide-1 (GLP-1) analogues
	or	Medication	NLM:ATC:A10BB	Sulfonylureas
Query Criteria for GLP-1				
Must have	-	Medication	NLM:ATC:A10BJ	Glucagon-like peptide-1 (GLP-1) analogues
	And	Diabetes Mellitus		
	And	Age is older than 50 years before the index event		
Cannot have	-	Medication	NLM:RXNORM:6809	Metformin
	or	Medication	NLM:ATC:A10BB	Sulfonylureas
	or	Medication	NLM:HS501	Insulin
Query Criteria for Sulfonylureas				
Must have	-	Medication	NLM:ATC:A10BB	Sulfonylureas
	And	Diabetes Mellitus		
	And	Age is older than 50 years before the index event		
Cannot have	-	Medication	NLM:RXNORM:6809	Metformin
	or	Medication	NLM:ATC:A10BJ	Glucagon-like peptide-1 (GLP-1) analogues
	or	Medication	NLM:HS501	Insulin
Query Criteria for Metformin Without DM				
Must have	-	Medication	NLM:RXNORM:6809	Metformin
	And	Age is older than 50 years before the index event		
Cannot have	-	Medication	NLM:HS501	Insulin
	or	Medication	NLM:ATC:A10BJ	Glucagon-like peptide-1 (GLP-1) analogues
	or	Medication	NLM:ATC:A10BB	Sulfonylureas
	or	Diabetes Mellitus		

Appendix 2

**Table 4:** Ethnicity, race, and gender identities of the patient cohorts that were balanced prior to comparative analyses TriNetX reports race, ethnicity, and gender data for patient cohorts as reported by the healthcare organizations based on patient-identified information.

Ethnicity	Race	Gender
Hispanic Patients	White Patients	Female
Not-Hispanic Patients	Alaska Native Patients	Male
Unknown Ethnicity Patients	Asian Patients	Unknown
-	Black Patients	-
-	Native Hawaiian Patients	-
-	Other Race Patients	-
-	Unknown Race Patients	-
